# 

**Published:** 1915-02

**Authors:** 


					﻿PUBLISHED MONTHLY.	ATLANTA	SUBSCRIPTION gj.OO
JOIUXAI-UK OPD
OF MI DICIXL
! Atlanta Medical and Surgical Journal, Established 1855. ) pi .
him Year
and Southern Medical Record, Established 1870.	julol l LG I
Vol. LXI.	Atlanta, Ga., February, 1915.	No. 11
				

